# Metabolite Profiling and Network Analysis Reveal Coordinated Changes in Low-N Tolerant and Low-N Sensitive Maize Genotypes under Nitrogen Deficiency and Restoration Conditions

**DOI:** 10.3390/plants9111459

**Published:** 2020-10-29

**Authors:** Arshid Hussain Ganie, Renu Pandey, M. Nagaraj Kumar, Viswanathan Chinnusamy, Muhammad Iqbal, Altaf Ahmad

**Affiliations:** 1 Department of Botany, Jamia Hamdard, New Delhi 110062, India; 10arshid@gmail.com (A.H.G.); iqbalg5@yahoo.co.in (M.I.); 2 Division of Plant Physiology, Indian Agricultural Research Institute, New Delhi 110012, India; renu.pandey.iari@gmail.com (R.P.); shastishanmuga@gmail.com (M.N.K.); viswanathan@iari.res.in (V.C.); 3 Department of Botany, Faculty of Life Sciences, Aligarh Muslim University, Aligarh 202002, India

**Keywords:** maize, GC–MS, LC–MS, metabolomics, nitrogen deficiency, nitrogen-use-efficiency, low-N tolerance

## Abstract

Nitrogen (N), applied in the form of a nitrogenous fertilizer, is one of the main inputs for agricultural production. Food production is closely associated with the application of N. However, the application of nitrogenous fertilizers to agricultural fields is associated with heavy production of nitrous oxide because agricultural crops can only utilize 30–40% of applied N, leaving behind unused 60–70% N in the environment. The global warming effect of this greenhouse gas is approximately 300 times more than of carbon dioxide. Under the present scenario of climate change, it is critical to maintain the natural balance between food production and environmental sustainability by targeting traits responsible for improving nitrogen-use-efficiency (NUE). Understanding of the molecular mechanisms behind the metabolic alterations due to nitrogen status needs to be addressed. Additionally, mineral nutrient deficiencies and their associated metabolic networks have not yet been studied well. Given this, the alterations in core metabolic pathways of low-N tolerant (LNT) and low-N sensitive (LNS) genotypes of maize under N-deficiency and their efficiency of recovering the changes upon resupplying N were investigated by us, using the GC–MS and LC–MS based metabolomic approach. Significant genotype-specific changes were noted in response to low-N. The N limitation affected the whole plant metabolism, most significantly the precursors of primary metabolic pathways. These precursors may act as important targets for improving the NUE. Limited availability of N reduced the levels of N-containing metabolites, organic acids and amino acids, but soluble sugars increased. Major variations were encountered in LNS, as compared to LNT. This study has revealed potential metabolic targets in response to the N status, which are indeed the prospective targets for crop improvement.

## 1. Introduction

Nitrogen (N) is essential for life sustainability. Some morphological, developmental and reproductive phenomena such as flowering, growth, senescence, oxidation, reduction and allocation of photosynthates in a plant are regulated by the availability of N [1]. Application of N fertilizers has both positive and negative impacts; it has increased the supply of food, feed and several biobased products remarkably on one hand, but also deteriorated the quality of the environment and caused huge economic losses by depleting the fossil-fuel reserves, on the other. Various forms of N released into the environment pose a serious threat to the health of humans, plants and animals [2]. Excessive use of non-sustainable fossil fuels results in heavy emission of greenhouse gases, causing depletion of the ozone layer, global warming and other serious environmental threats. The rapid increase in human population demands more agricultural production, which is achievable by using heavy nitrogenous fertilizers. However, agricultural crops, particularly rice, maize and wheat, have an N-utilization efficiency of only 30–40%, leaving behind 60–70% N unused, which severely deteriorates the environmental health [3]. It is assessed that the anthropogenic contribution to greenhouse gas production from agricultural fields is around 10–12% of the total greenhouse gas (GHG) emissions resulting from human activities [4]. The main source responsible for agricultural releases results from the manufacture and excessive application of nitrogenous fertilizers to cultivable land. Synthetic production of N fertilizers employing the Haber–Bosch reaction accounts for about fifty percent of the total energy expenditure in agriculture [5]. Moreover, application of nitrogenous fertilizers to agricultural fields is associated with heavy production of nitrous oxide. The global warming effect of this greenhouse gas is approximately 300 times greater than that of carbon dioxide [6] and constitutes about forty percent of GHG emissions directly from agricultural soils [4]. It is, therefore, important to limit the application of nitrogenous fertilizers without affecting the crop yield [3]. Hence, a clear rationale has to be defined, emphasizing on the reduction of the excessive utilization of N fertilizers in the agriculture sector.

The targets for future research include development of a highly productive agriculture to increase crop productivity, coupled with a reduction in the use of N fertilizers [7]. An appraisal by the FAO has shown that there is a need to increase agricultural production by 60% in 2030–2050 over the production levels in 2005–2007 [8]. In India, maize is the third most important food crop after rice and wheat, and consumes large quantities of nitrogenous fertilizers [9]. The average nitrogen-use-efficiency (NUE) is far less than 50% and, therefore, enhancing the NUE is the best approach in majority of crops, specifically in those that require huge quantities of N fertilizers for a maximum yield [10]. Few agronomic methods such as the slow release of fertilizers, nitrification inhibitors and split application of N are used to get the maximum benefits of applied fertilizers. The conventional breeding is also used to select the most appropriate traits, but this practice provides no information on the molecular basis of enhancement in NUE. Various studies, including the whole plant physiology, agronomy and molecular genetics, have been undertaken over the last two decades for characterizing the switches that regulate the NUE. Some genomic studies have indicated that overexpression of transcription factor *DOF1* under low-N conditions results in increased plant growth and N content [11]. All such studies have been based on a particular gene, protein or trait; however, the NUE may depend on the interaction of a network of genes. Therefore, a holistic approach that considers all the genes and their associated pathways for improving NUE is the need of the hour.

To address the root causes that regulate the NUE, some studies have used metabolomic, transcriptomic and proteomic approaches [12,13,14]. The physiological status of a cell, tissue and organ is recorded through the ‘omics’ procedure at different developmental stages of the plant [15]. This offers a complete overview of alterations in the concentration of metabolites (primary and secondary), gene transcripts and proteins [16,17]. An untargeted metabolite profiling, covering the entire range of metabolites, must be helpful in developing strategies to improve NUE and in collecting information about gene products or expression of new genes [18]. Metabolomics provides complete information about what is happening inside the tissue, organ or a cellular compartment of plant under various stresses caused by adverse factors, physiological adaption to dietary change or environmental perturbations [19,20,21,22,23,24]. It has also been applied to studies related to shortage in nutrients [25]. Earlier studies on metabolic responses of maize to N generally concentrated at the vegetative and maturation stages of leaves only [26]. These studies did not include the resupply condition, which is important to validate whether the effect is due to the factor under observation or to some other reasons. Further, metabolomic data of low-N tolerant and low-N sensitive maize, involving leaf, roots and the effect of resupply of N at different time intervals still remain uncovered. Given this, the present investigation was conducted to analyze the non-targeted metabolic profiling of shoot and root of two contrasting maize genotypes (low-N tolerant and low-N sensitive) in response to N-deficiency and resupply of N. This could help in explaining the key role of master switches/regulators, responsible for the diverse N responsiveness of different genotypes of the same species.

## 2. Results

### 2.1. Growth and N Status of Maize Genotypes under N Deficiency

The low-N tolerant (LNT) and low-N sensitive (LNS) maize plants were grown either with low-N (0.05 mM, −N) or with sufficient-N (4.5 mM, +N, control) supply in the nutrient solution. After fifteen days of growth in Hoagland’s solution, the root length of LNS plants increased significantly, while shoot growth was highly reduced under low-N supply, as compared to the control (sufficient-N supply). However, no such significant change was noticed in the LNT genotype in shoot and root length under N-limiting conditions (Table 1, Figure S1). Biomass accumulation was drastically reduced in the LNS genotype under low-N conditions, compared to the control (sufficient-N supply). Slight significant reduction of biomass accumulation was observed in the LNT genotype under the low-N condition. Rate of photosynthesis and chlorophyll content were significantly reduced in the LNS genotype, while LNT showed no significant difference under similar environments and conditions. The N concentration in leaves of maize genotypes clearly showed that the N status was reduced under N-limiting conditions in both the genotypes. However, the reduction of the N concentration was lesser in the LNT genotype than in the LNT genotype (Table 1). This is why the low-N conditions did not influence the root and shoot biomass of the LNT genotype. Furthermore, the LNT maize genotype maintained the efficiency of utilizing N sources under low-N conditions, however LNS was unable to maintain balance between the source and sink under N-limiting conditions. This shows that the LNT genotype has the ability to acclimatize with the alteration in the nutritional status and, therefore, has the potential to grow better in soils low in N. The variable response of LNT and LNS genotypes to N deficiency indicated a true physiological response to N-deficit. Therefore, any change that may occur at the metabolite level may be logically correlated in plants grown under low-N conditions.

### 2.2. Principal Component Analysis (PCA) of Metabolites of Maize Genotypes

**Figure 1:**
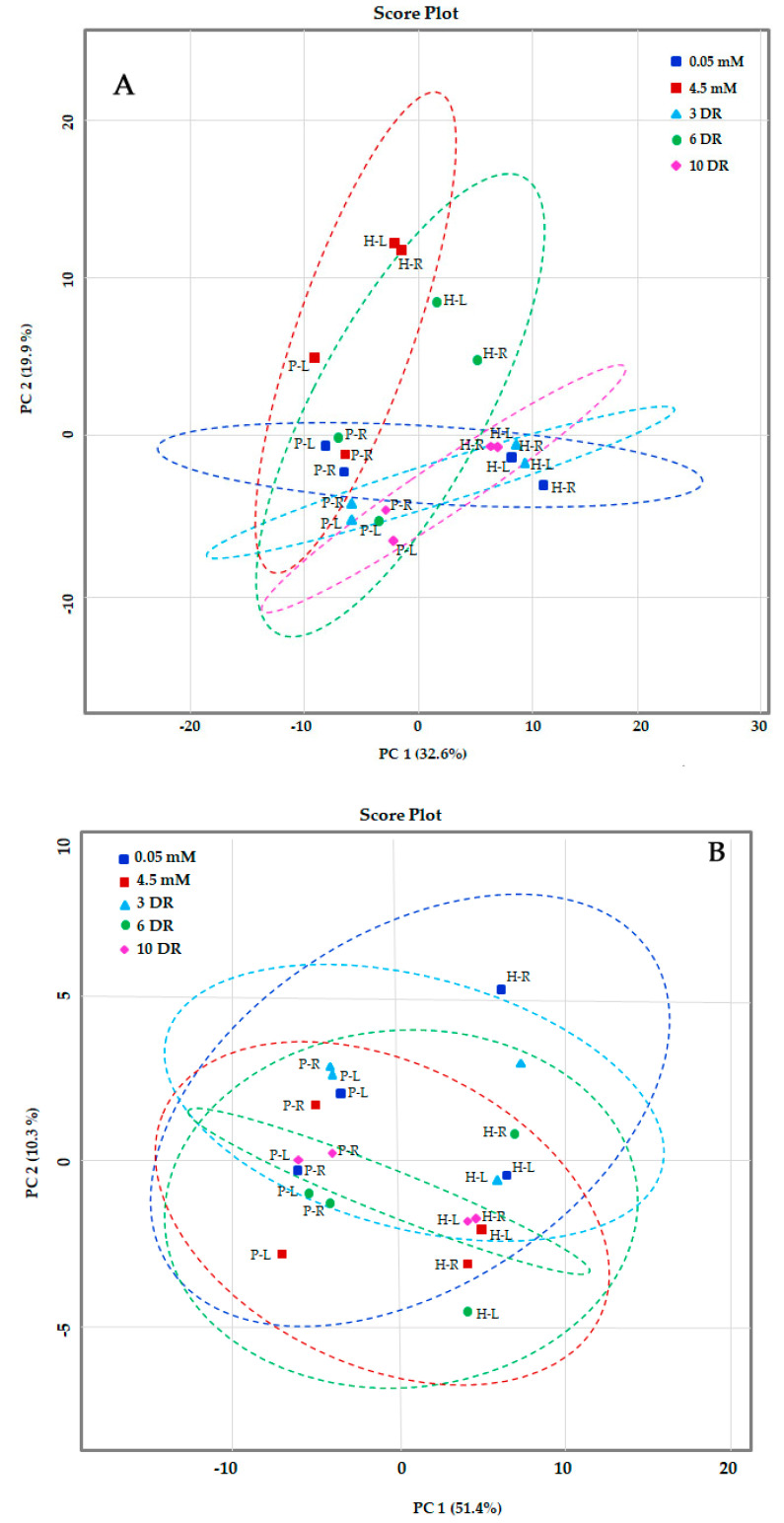
Principal component analysis of the GC–MS (**A**) and LC–MS (**B**) based metabolic profile of the leaf and root of PEHM-2 and HM-4 maize genotypes under low-N (0.05 mM) and its recovery and sufficient-N (4.5 mM) conditions. The principle component analysis (PCA) score plot distinguishes the metabolic profiles of low-N sensitive and low-N tolerant maize genotypes. Legend for variables: PEHM-2 leaf (P-L), PEHM-2 root (P-R), HM-4 leaf (H-L), HM-4 root (H-R), 0.05 mM N (blue filled square), 4.5 mM N (red filled square), 3DR (sampling after 3rd day of resupply N, triangle), 6DR (sampling after 6th day of resupply N, circle) and 10DR (sampling after 10th day of resupply N, diamond). The first number in the data points represents treatment (0.05 mM N, 4.5 mM N, 3DR, 6DR and 10DR). The second number represents the genotype (P and H). The third number indicates the plant organ (leaf and root).

**Figure 2:**
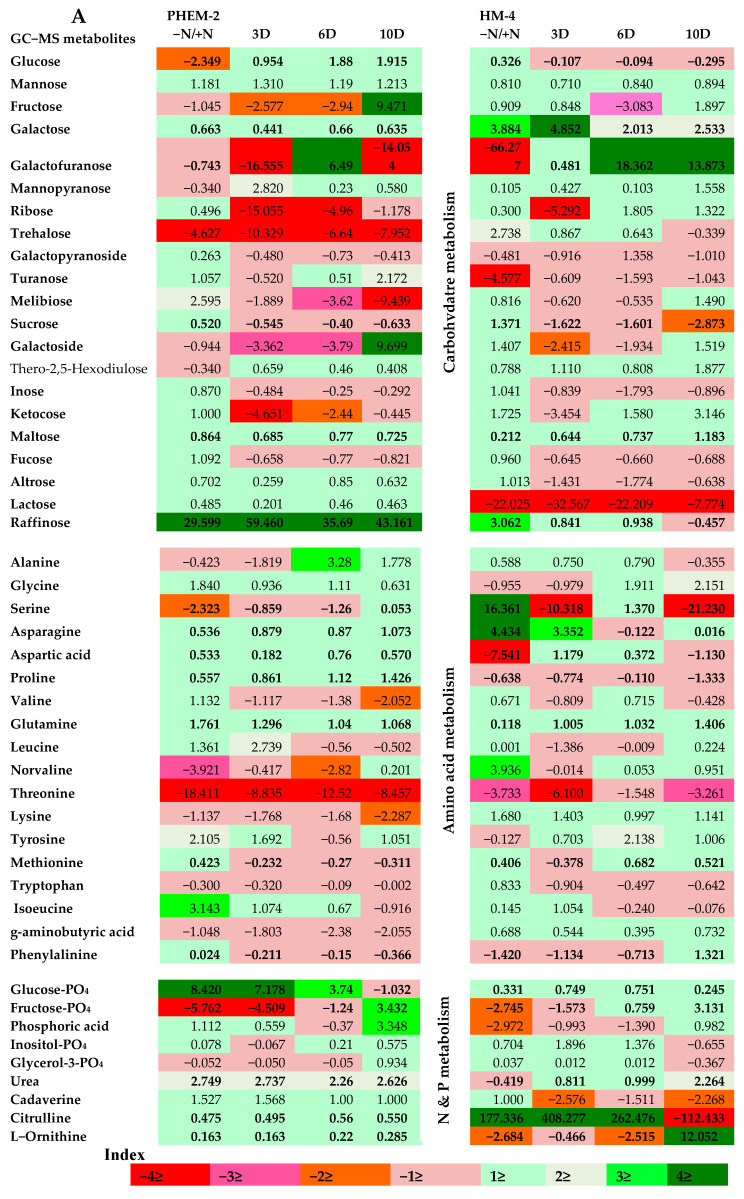

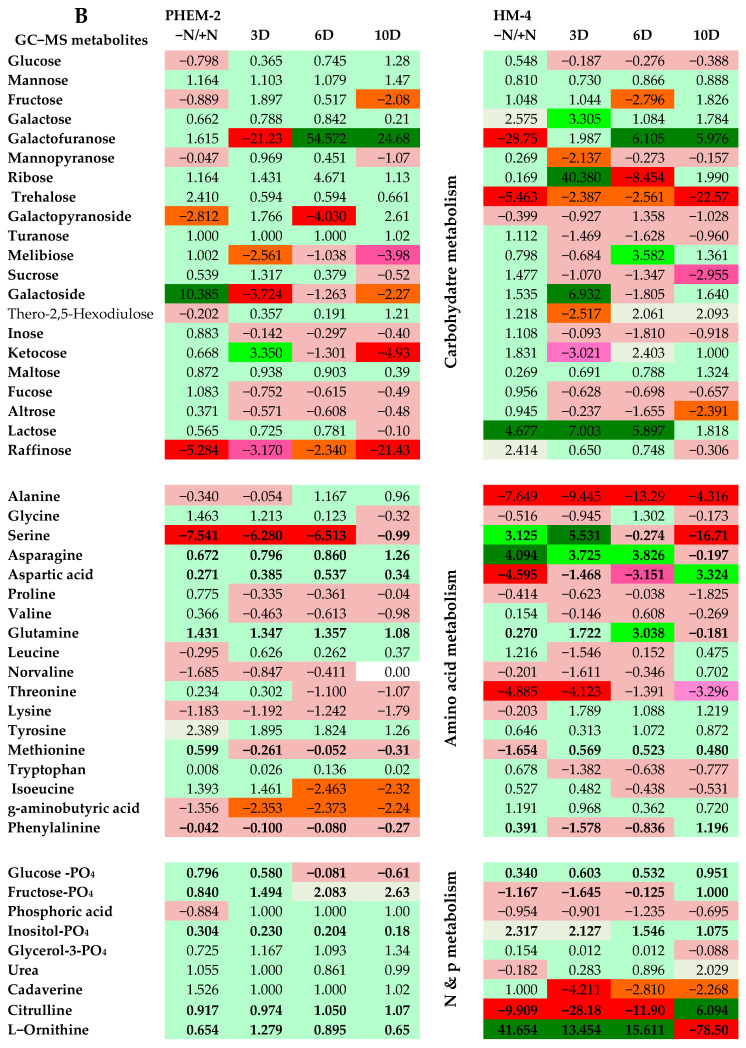
Metabolic responses to N deficiency of low-N tolerant (LNT; PEHM-2) and low-N sensitive (LNS; HM-4) genotypes. (**A**) Root metabolites and (**B**) leaf metabolites. The calculated data shown above exhibit a log transformed fold change (0.05 mM/4.5 mM N) of selected metabolites detected by GC–MS in low-N (0.05 mM), sufficient-N (4.5 mM) and recovery (3DR, 6DR and 10DR) samples. The level of significance between sufficient-N (4.5 mM) and low-N (0.05 mM) treatments was tested by the Student’s *t*-test (*p*-value < 0.05). Significantly changed metabolites in various groups are represented in bolded form. Upregulation and downregulation of metabolites are indicated with the help of color bar scale, upregulated (green) or downregulated (red), in metabolite logarithmic fold change, as indicated in the color index (*n* = 6).

**Figure 3:**
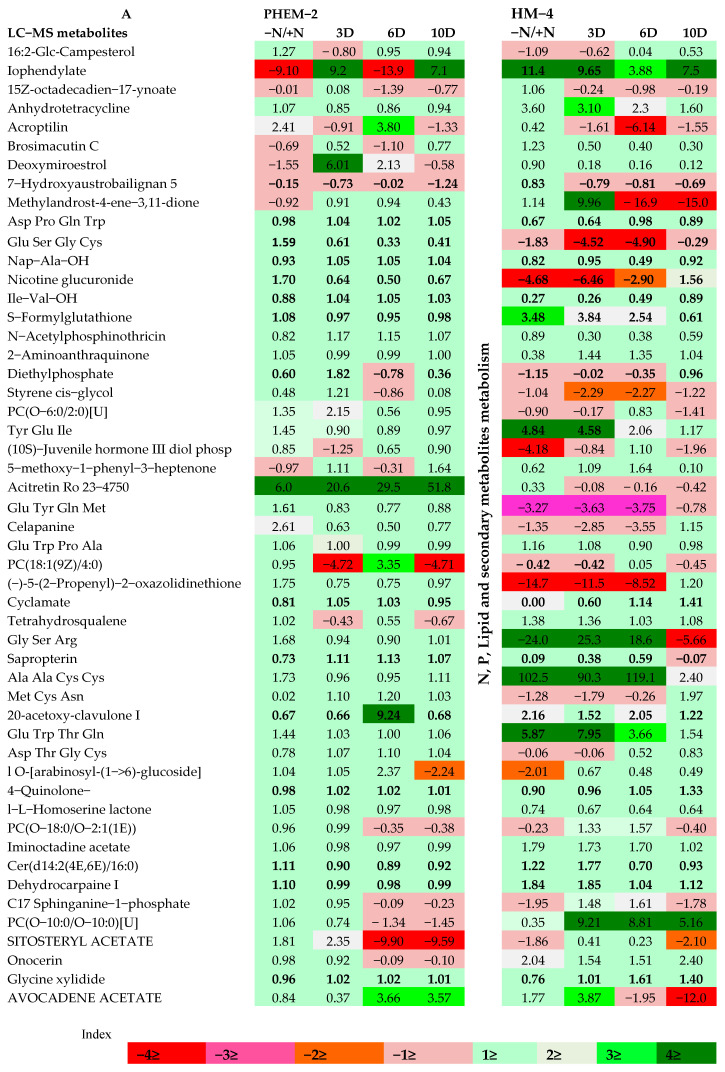

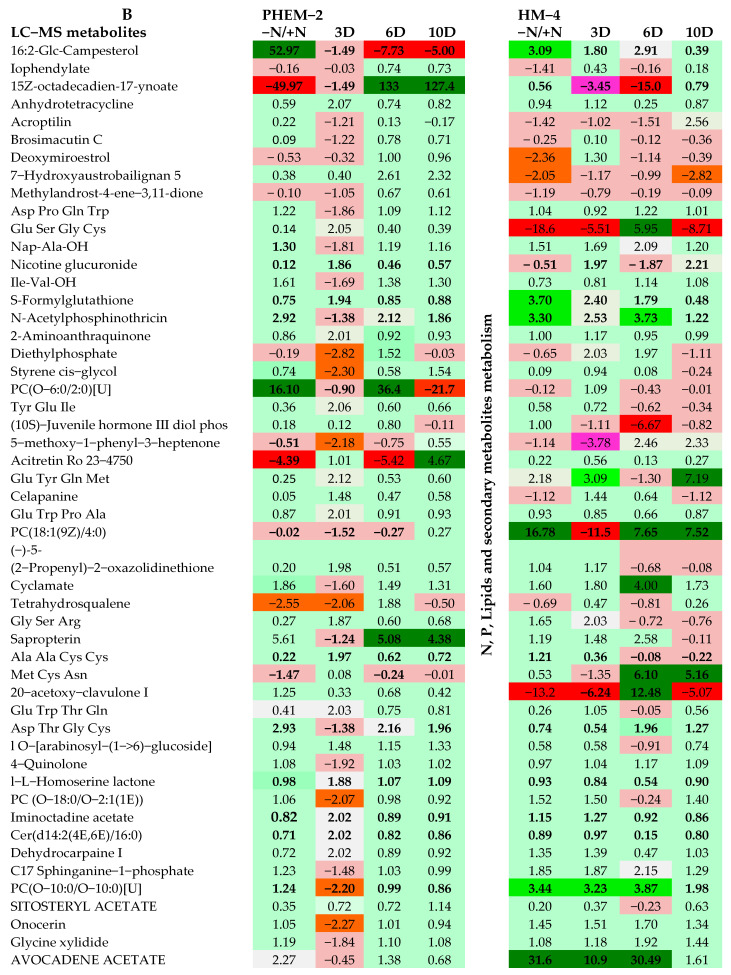
Metabolic responses to N deficiency of low-N tolerant (LNT; PEHM-2) and low-N sensitive (LNS; HM-4) genotypes. (**A**) Root metabolites and (**B**) leaf metabolites. The calculated data shown above exhibit a log transformed fold change (0.05 mM/4.5 mM N) of selected leaf metabolites detected by LC–MS in low-N (0.05 mM), sufficient-N (4.5 mM) and recovery (3DR, 6DR and 10DR) samples. The level of significance between sufficient-N (4.5 mM) and low-N (0.05 mM) treatments was tested by the Student’s *t*-test (*p*-value < 0.05). Significantly changed metabolites in various groups are represented in the bolded form. Upregulation or downregulation of metabolites is indicated with the help of color bar scale, upregulated (green) or downregulated (red), in the metabolite logarithmic fold change, as indicated in the color index (*n* = 6).

Metabolome of LNS and LNT genotypes was analyzed using LC–MS and GC–MS to examine the effect of N deficiency and restoration of N supply on the differential levels of the metabolites in leaves and roots. Metabolites were profiled from leaves and roots of LNS and LNT genotypes growing under conditions of low-N (0.05 mM N), sufficient-N (4.5 mM N) and resupply of N (3DR, 6DR and 10DR). The morphological variations, such as the reduced shoot growth, color intensity and increased root length, were more prominent in LNS than in the LNT genotype. Among the identified peaks, a total of 94 and 57 metabolites were found in the leaves and roots of maize by GC–MS and LC–MS, respectively. Libraries available in the database linked to the GC–MS and the LC–MS instruments enabled the putative identification of compounds associated with each peak. Principle component analysis (PCA) was performed to figure out specific data patterns from the complex data sets. A specific metabolic pattern elicited by the deficiency of nitrogen in low-N tolerant (PEHM-2) and low-N sensitive (HM-4) maize genotypes was observed. The samples, which are related, were clustered together after plotting different sample groups in space resulting from those variables (main components) responsible for separation of the whole dataset. Based on the degree of similarity and dissimilarity between samples, five ellipses of the data were generated in the score plot. The scores of the analyses revealed a clear characteristic metabolic profile of the leaf and root of PEHM-2 and HM-4 based on the metabolite positions in the 2-D plot (Figure 1A,B). The data of the metabolic profile of the leaf and root of PEHM-2 and HM-4 under the nitrogen-sufficient condition resembled the conditions of N-restoration at the 3rd, 6th and 10th day, because sufficient-N treatment and recoveries overlapped each other, confirming that changes occurring in the plants’ development were due to the targeted macronutrient. The low-N grown HM-4 (leaf and root) fell apart. The data also revealed that the effect of low-N stress was more in HM-4 than PEHM-2. It is also observed from the graph that the metabolic profile of samples taken from deficient N containing media were more distant from each other, as compared to PEHM-2. The metabolic trend revealed by PCA was highly similar in the leaf, root and restoration samples but highly variable from treated samples (Figure 1A,B). The score plot of the GC–MS and LC–MS based metabolite profiling showed that the first principle component (PC1) represented 32.6% and 51.4% variation, respectively, which were experiment-specific variations (Figure 1A,B, Table S1). The principal component vectors, PC1 and PC2, accounted for 52.5% of the total variation under low-N conditions. Principle component analysis (PCA) based on GC–MS and LC–MS indicated the separation of LNS and LNT genotypes along PC1 (32.6% of data variance) and 19.9% along PC2. This observation is mainly attributed to the secondary metabolites like (bromo-3-hydroxy-4-(succin-2-yl)-caryolane, 15z-octadecadien-17-ynoate, cyclamate, sapropterin, 20-acetoxy-clavulone I, l o-arabinosyl-(1->6)-glucoside, l-L-homoserine lactone and organic acids such as PC(O-10:0/O-10:0)[U]. Surprisingly, coherence was found between the metabolite profiling based on LC–MS and GC–MS, showing that N deficiency led to the separation between the genotypes (Figure 1A,B). The LC–MS data subjected to random forest (RF) identified metabolic markers for nutrition deficiency. Random forest generated a parabolic plot picturing the degree of the impact responsible for the separation between low-N, sufficient-N and various restoration treatments. Consequently, the effect of a shortage of N was observed in LNS on day 15 of the experiment as reflected by the parabolic-shaped data distribution. Among the identified markers, the analysis particularly highlighted Ile-Val-OH, Sapropterin, PC (O-10:0/O-10) and Celapanine as being highly affected by the limited availability of N in LNS (Table S2, Figure S2). The whole normalized values of metabolite data sets were presented in the form of heatmaps. The relative concentration of metabolites of plants under low-N conditions (0.05 mM) and the N-sufficient (4.5 mM) condition was calculated as a response ratio (0.05 mM/4.5 mM). The 0.05 mM/4.5 mM ratios for metabolite levels with statistically significant increases or decreases of at least 33% are summarized in Figure 2 and Figure 3.

### 2.3. Metabolite Profiling of the Leaf and Root of Maize Genotypes under N Treatments

This study elucidated the metabolites profiling in roots and leaves of maize genotypes under nitrogen starving conditions. Low molecular weight, volatile and thermally stable compounds were profiled with the aid of GC–MS. The shortcomings of the GC–MS were overcome by the LC–MS that provides a broad overview of high molecular weight, polar and thermally less stable compounds.

Major metabolites of carbon and nitrogen assimilation that contribute to enzymatic reactions exhibited significant differences under conditions of N sufficiency (4.5 mM), N deficiency (0.05 mM) and N restoration (Figure 2A,B). Ninety four putative identified metabolites showed significant differences in the three replicates (*p* ≤ 0.05). Among these metabolites, the relative content of the majority of the amino acids (total detected 18) decreased. For instance, serine showed the highest reduction (7.4 fold) in the leaf and a relatively less reduction (2.3 fold) in the root of LNT, whereas in LNS, it increased by 3.1 fold in the leaf and to the maximum (16.3 fold) in the root under low-N conditions (0.05 mM). Other amino acids were also reduced in the LNS genotype under low-N conditions. The reduction of alanine, aspartic acid and threonine were 7.6 fold, 4.5 fold and 4.8 fold, respectively, in leaves. Similar observations were made in roots except for aspartic acid that showed the maximum reduction (7.5 fold) in the LNS genotype. On the contrary, such reductions were not found in the LNT genotype under low-N conditions (Figure S3). Several N-containing metabolites such as urea, uridine and citrulline (9.9 fold) in the leaves significantly increased, whereas ornithine (41.6 fold) and citrulline (177.3 fold) in the leaves and roots of LNS significantly increased under low-N conditions. Not many changes were observed in N-containing metabolites of LNT; however, small reductions were observed in γ-amino butyric acid in leaves and roots of the LNT genotype under low-N conditions. Apart from this, a significant decrease was recorded in the amounts of organic acids, such as those taking part in tricarboxylic acid (TCA) and C_3_, C_4_ carbon metabolism and the β-oxidative pathway, analogous to the catabolism of fatty acids, remarkably benzoic acid (8.8 fold) in the roots of the LNT genotype (Figure 2A,B; Figure S3). Maleic acid was also reduced (4.3 fold) in the leaves and roots of the LNS genotype. However, there was a significant increase in mannonic acid (99.3 fold) in leaves and 32.6 fold in roots of the LNS genotype. In addition to this, parabanic acid (11.02 fold) was significantly reduced in the roots of the LNT genotype. Under low-N (0.05 mM N) conditions, the amount of most of the soluble sugars (glucose, fructose, mannose, galactose and raffinose) was reduced in LNT roots and leaves whilst such changes were contradictory to the LNS genotype except for galactofuranose and trehalose that were significantly reduced by 28.7 fold and 5.4 fold, respectively, under low-N conditions (Figure 2A,B; Figure S3). Phosphate-containing sugars like glucose-PO_4_ and fructose-PO_4_ were significantly reduced under the low-N condition in case in both leaves and roots of the LNS genotype under low-N conditions (Figure 2A,B; Supplementary Figure S3). In contrast, LNT shows some degree of tolerance to this change, though glucose-PO_4_ increased significantly (8.4 fold) in roots under low-N conditions (Figure 2A,B). Polyamines like cadaverine also decreased under low-N conditions in the case of the LNS genotype. The relative content of sugar alcohols such as erythritol and mannitol contrastingly increased in the roots and leaves of LNS genotypes under low-N conditions, whereas galactinol was reduced. In comparison to this, the erythritol and mannitol contents were reduced while the galactinol content increased in the LNT genotype under low-N conditions (Figure 2A,B). Phytosterols, like stigmosterol (3.8 fold), were significantly reduced in both the roots and leaves of LNS, whereas LNT was relatively stable in this regard (Figure S3).

The narrow range metabolite coverage of GC–MS based metabolite profiling was overcome by more sensitive LC–MS based metabolite fingerprinting, which showed that alterations in the metabolism under progressive N deficiency were genotype specific. The impact of the low-N condition on high molecular weight mid-polar and non-volatile metabolites (thermally less stable) was determined with the help of LC–MS. The snapshot of secondary metabolism indicates milder changes in metabolites of the secondary metabolic pathways, compared to those involved in the central metabolic pathways, and these were higher in the LNS genotype than in the LNT genotype (Figure 3A,B). Furthermore, the VIP plot on the LC–MS dataset, which included a number of markers, emphasized that secondary metabolites (7-hydroxyaustrobailignan, iminoctadine acetate, PC (O-10:0/O-10, raphanusamide and sapropterin)) were the main contributors to variations between treatments. Levels of flavones such as brosimacutin C and deoxymiroestrol and the tri-peptide, glutathione, increased significantly under low-N conditions (Figure 3A,B).

The main metabolites of major enzymatic reactions, including the carbon and nitrogen assimilations, showed significant differences under sufficient-N (4.5 mM), low-N (0.05 mM) and restoration conditions (Figure 3A,B). Among the 154 detected peaks, 57 metabolites were detected as known metabolites with the aid of the METLIN tandem database (Scripps Center for Metabolomics; Table S3). Among the putatively identified metabolites some of the lipids and N-containing metabolites changed significantly under low-N conditions in the LNS genotype, but little changes were observed in the case of the LNT genotype. Metabolites that decreased significantly include bromo-3-hydroxy-4-(succin-2-yl)-caryolane (4.2 fold), acroptilin (1.4 fold), 7-hydroxyaustrobailignan (2.05 fold), Glu Ser Gly Cys (18.6 fold), celapanine (1.2 fold) and 20- acetoxy-clavulone I (13.3 fold) in the leaves, while in the roots such changes were not detected except that nicotine glucuronide (4.6 fold), Glu Ser Gly Cys (1.83 fold), 5-(2-propenyl)-2-oxazolidinethione (14.7 fold) and (10S)-Juvenile hormone III diol phosphate (4.1 fold) were reduced under low-N conditions in the LNS genotype (Figure 3A,B). In contrast to this, only slight changes were observed in the metabolites of LNT leaves and roots under low-N conditions, showing the tolerance capacity of this genotype to low-N conditions. Besides, the metabolites that increased significantly in the leaves of the LNS genotype under low-N conditions include S-formylglutathione (3.7 fold), N-acetylphosphinothricin (3.3 fold), 16:2-Glc-campesterol (3.0 fold), PC (18:1(9Z)/4:0) (16.7 fold) and avocedene acetate (31.6 fold), whereas the major metabolites whose relative content increased in roots include anhydrotetracycline (3.6 fold), Tyr Glu Ile (4.8 fold), Gly Ser Arg (24.0 fold), Ala Ala Cys Cys (102.5) fold and avocedene acetate (17.7 fold). On the contrary, no such significant changes were detected in the metabolite profile of leaves and roots of LNT genotype leaves with the exception of 16:2-Glc-campesterol and PC (O-6:0/2:0)[U] that showed a significant increase of 52.9 fold and 16.1 fold, respectively, in the leaves of LNT under low-N conditions (Figure 3A,B).

### 2.4. Pathway Network and MESA Analysis

**Figure 4:**
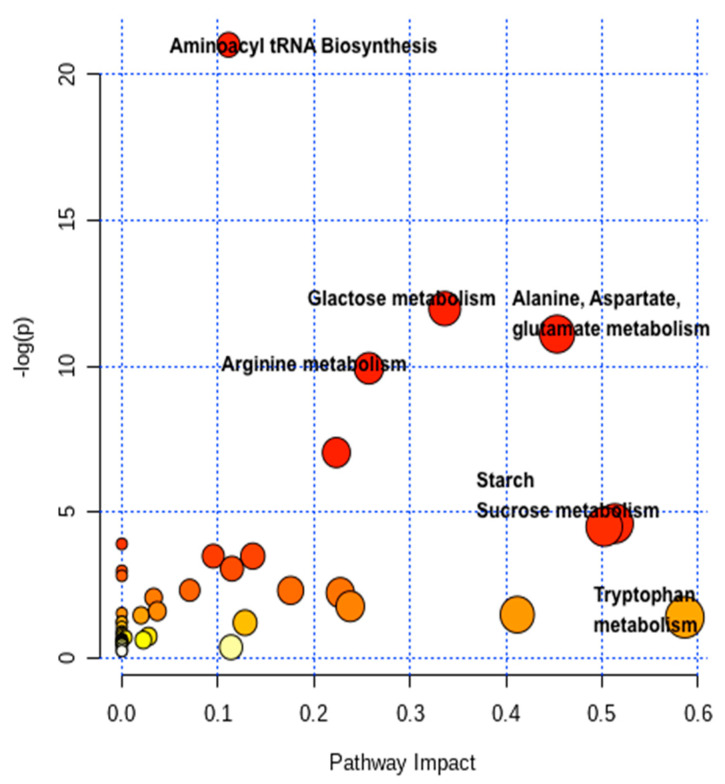
Metabolic pathway analysis plot created by using the MetaboAnalyst 2.0 software. Plots depict a change in several metabolic pathways due to the conditions of low-N, sufficient-N and restorations of N supply (3rd, 6th and 10th day) in maize genotypes. The x-axis represents the pathway impact values computed from the pathway topological analysis, and the y-axis is the -log of the *p*-value obtained from the pathway enrichment analysis. The color and size of each dot were associated with the -log (*p*) value and pathway impact value, respectively, where a small *p* value and high pathway impact value indicate that the pathway is greatly influenced.

To identify the pathways that are affected by the conditions of low-N, sufficient-N and restoration of N supply in the leaves sand roots of maize genotypes, metabolite profiles were analyzed using the MetaboAnalyst 2.0 software, which is a web-based software that derives its predictive ability from the Kyoto Encyclopedia of Genes and Genomes (KEGG) metabolic pathways database. It utilizes pathway enrichment and the topology analysis to identify pathways that are most significantly perturbed under the specific experimental conditions. Our analysis identified several pathways, which were significantly affected by N deficiency and had a major impact on the overall metabolic adjustments of plants. While Figure 4 presents a summary of the pathway analysis, the actual results are shown in Table 2. The total number of compounds that participated in specific pathways such as galactose metabolism (27), aminoacyl-tRNA biosynthesis (46), cyanoamino acid metabolism (26), glycine, serine and therionine metabolism (33), starch and sucrose metabolism (22), arginine and proline metabolism (28), alanine, aspartate and glutamate metabolism (22) and glutathione metabolism (27), indicates that amino acid metabolism is highly affected due to N starvation. To determine whether these alterations in the metabolomic profile of leaves and roots are related to low-N availability, a similar study was conducted on the same genotypes by resupplying N (restoration). It was observed that the changes that were caused by low-N deficiency in various metabolic pathways, such as serine, N-containing metabolites, organic acids levels and several metabolites, got eliminated and normal levels were restored in LNT roots and leaves. Contrary to this, the efficiency of restoration was very poor in the case of the LNS genotype. The LNS genotype showed a variable response to restoration as some metabolites got instantly increased such as proline, threonine and alanine after the 3rd day recovery, then again declined after the 6th day, thus depicting the extent of sensitiveness of LNS to the non-availability of N. The coherent results were obtained with the aid of LC–MS, which showed that the changes taken place due to low-N deficiency in various metabolic pathways such as those of fatty acids, N-containing metabolites, organic acids, etc., are restored to normal levels in leaves and roots of the LNT genotype. In contrast, the efficiency of restoration was very poor in the LNS genotype. Analysis of restoration samples of LNS genotype showed the available response to restoration, as some metabolites were instantly increased. The contents of Asp, Thr, Gly, Cys, celapanine, Glu, Tyr, Gln and Met increased after the 3rd day recovery, then went down after the 6th day, thus showing the extent of sensitiveness of the LNS genotype to the non-availability of N. In general, very few compounds like xylitol, sorbitol, ethanodic acid, malonic acid, asparagine and glutamine got accumulated under low-N conditions.

## 3. Discussion

**Figure 5:**
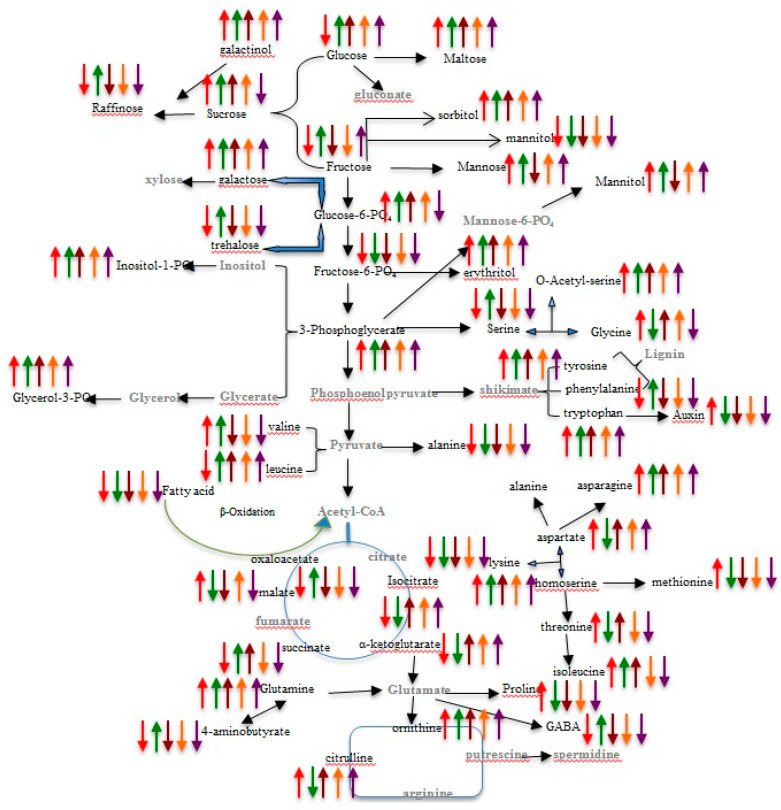
Comparative changes in the metabolites involved in primary metabolic pathways of leaves and roots of LNT (PEHM-2) and LNS (HM-4) genotypes in response to N deficiency. The detected and undetected metabolites are represented by black and grey notation respectively. The red and green arrows indicate LNT and LNS genotypes, respectively, while arrows in brick red, light yellow and purple indicate the restoration samples at the 3rd, 6th and 10th day (3DR, 6DR and 10DR) respectively. The metabolites found to have upregulated or downregulated under low-N conditions are represented by upward-pointed and downward-pointed arrows respectively.

In this study, an effort was made to profile metabolites of leaves and roots of two contrasting genotypes, PHEM-2 (low-N tolerant, LNT) and HM-4 (low-N sensitive, LNS) of maize, growing under low-N and restoration conditions. The study has provided a comprehensive and comparative analysis of the metabolite composition in leaves and roots of contrasting maize genotypes under conditions of low-N and restoration of N supply. When data from LNT and LNS genotypes were combined, leaves and roots of LNT had the most similar metabolite content to the associated control (4.5 mM N), whereas in the case of the LNS genotype, significant variations were detected within treatments, i.e., low-N 0.05 mM N and its control 4.5 mM N, and between genotypes. Out of 130 detected peaks, 94 metabolites were putatively identified as known compounds based on the mass spectra library NIST, Wiley Registry, Golm and Fiehn database. Most of the amino acids except glutamine, asparagine, glycine and the N-containing metabolites were significantly reduced under low-N conditions. Serine was significantly reduced under low-N conditions, as was reported earlier by Rossella et al. [27]. This might be due to the reduction in the ATP-sulphurylase and O-acetylserine sulphydrylase activities under N-deprivation. Proline and alanine were also reduced significantly under low-N conditions. An increase in the content of proline and alanine could serve as an indicator of an imbalance in N nutrition [28]. Aspartic acid and glutamate were also reduced under low-N conditions, as they may be involved in the formation of oxalacetate, an important intermediate of the tricarboxylic acid cycle, as assimilation of NH_4_^+^ into amides and amino acids requires carbon skeletons from the tricarboxylic acid cycle [29]. This could be why there is a general decrease in the organic acid content, as there is a shortage in the amount of precursor molecule. The main regulators of the amino acid biosynthesis are 2-oxoglutarate and glutamate. The level of these metabolites increased, following a transfer to the N-sufficient medium [30]. Our study substantiated these findings. There was a general reduction in the levels of various amino acids under low-N condition. There was a contrast between γ-amino butyric acid contents of leaves and roots in LNS and LNT genotypes; it accumulated in the LNS genotype and declined in the LNT genotype under low-N conditions. The level of this amino acid may control the interaction between assimilatory pathways of N and C and photorespiration [31]. Accumulation of GABA was seen in our study under the low-N condition. Synchronized regulation of GABA in plants has also been reported earlier [32]. Other amino acids that accumulated under low-N conditions include asparagine, glutamine and glycine. Accumulation of glutamine and asparagine could be related to the remobilization of assimilated N. Glutamine is not only used for N transport but also serves as an amino donor to other amino acids. Previous studies have shown that N-containing macromolecules and C reserve compounds like carbohydrates and fats are accumulated under N-starvation conditions [33,34]. Most of the N-containing metabolites, such as urea, citrulline, celapanine, sapropterin, anhydrotetracycline, 2-aminoanthraquinone and dehydrocarpaine I, were reduced under low-N conditions, possibly to conserve nitrogen for important developmental processes. It has been suggested earlier that the reduced concentration of urea might owe to the slow catabolism of arginine in the urea cycle in the N-deficient plants [35]. Our study also revealed a reduced concentration of allantoin under low-N conditions. Allantoin is involved in storage, translocation and signaling of N [36,37]. That plants save nitrogen by reducing the N-containing metabolite levels was also proposed by Lu and Zhang [38]. It has been reported that there is conservation of N by the lessening synthesis of proteins and chlorophyll in low-N grown plants. Since, N deficiency in plants alter mainly nitrogen and carbon metabolism, we attempted to integrate different primary and secondary metabolic pathways at important metabolic switches. These metabolic switches function as precursors and regulate the development and yield of crops. Therefore, giving an integrative picture of the metabolic network affected by nutrition deficiency may pave the way for future studies to enhance nutrient use efficiency and yield of crops (Figure 5). Apart from the decreases in various N-containing metabolites and peptides, the level of glutathione, a tri-peptide, was increased significantly in leaves and roots of the LNS genotype under low-N conditions, while no significant change observed in the LNT genotype (Figure 5). Glutathione protects the plasma membranes by maintaining the level of α-tocopherol and zeaxanthin. Since, membranes are more vulnerable to environmental stresses, maintenance of the proper structure of biomembranes is an essential requirement.

Lipids contents, e.g., linolenic acid, linoseaure 16:2-Glc-Campesterol, PC (O-6:0/2:0)[U] and sitosteryl acetate, increased significantly under low-N conditions, possibly because plants tend to accumulate lipids as an alternate source of energy. Metabolism slows down under low-N conditions, as lipids are slowly broken to release energy. Earlier, accumulation of carbon metabolites as lipids was reported under nitrogen deficient conditions in algal cells [39]. Sterols are also the important components of biomembranes; any alteration in their composition will produce drastic effects in the development of the plant. This study has revealed that lipid and sterol, like 16:2-Glc-Campesterol and sitosteryl acetate increased in the LNT genotype; while opposite was the case in the LNS genotype under low-N conditions (Figure 5), because the LNS genotype was not able to acclimatize to low-N conditions. In general, plant sterols, such as stigmosterol, accumulated in response to low-N conditions. Current evidence indicates that plant sterols are proficient to modify the activity of the plasma membrane H^+^-ATPases [40]. It was shown that the sterol modulation of the plasma membrane H^+^-ATPase activity depends on both the concentration and molecular species of sterol. One of the functions of ATPase is the production of the proton motive force across the plasma membrane, which is essential for the transport of ions and metabolites. The reduced biomass observed in the case of the LNS genotype under low-N and restoration confirmed the effect of a change of H^+^-ATPase activity (cell growth). Altered signaling of auxin and ethylene shown by *hyd* mutants could be elucidated by such effects [41]. As the alteration in auxin and ethylene signaling will affect the exudation of carbohydrates, amino acids and organic acids, it is an important strategy plants utilize for obtaining the locked nutrients from the soil under nutrient-limiting conditions, because these organic acids solubilize the rhizosphere in order to release elemental nutrients from bound forms. Consistently increased exudation of organic acids has been reported earlier under the conditions of low P and low Fe [42,43]; however, a lower root exudation of carboxylates, sugars and amino acids has been detected in N-deficient bean plants [44]. The regulatory switches behind this strategy remain unclear and maybe plant growth regulators, for instance indol-acetic acid (IAA) and zeatin, play a role. Comparative study of the H^+^-ATPase activities at the cell membrane under various nutrient regimes may shed further light on the significance of retrieval mechanisms for the net exudate discharge from roots. Cholesterol has been reported to act as a signaling molecule in the animal system, without the transformation into steroid hormones [45]. Such a function can also be assumed for plant sterols. Under low-N conditions, in our study, monoacyl and tri-acyl glycerols such as PC (O-6:0/2:0)[U], PC [O-18:0/O-2:1(1E)], Cer(d14:2(4E,6E)/16:0) and PC (O-10:0/O-10:0)[U] accumulated. These results endorse earlier findings with *Chlamydomonas reinhardtii* that builds up both starch and TAGs in response to various types of stresses including N deprivation [46,47,48,49,50,51,52,53,54,55,56].

Under low-N conditions, there was a dramatic reduction in the levels of major organic acids of the tricarboxylic acid cycle, particularly ketoglutaric acid, succinate, isocitric acid and malic acid. The findings were in accordance with those of Scheible et al. [57] who reported that the limited N supply lead to large decreases in 2-oxoglutarate, isocitrate and malate. Organic acids are the preferred source of carbon under nutrient-limiting conditions. Additionally, phosphate-containing sugars (glucose-PO_4_ and fructose-PO_4_) are significantly reduced under low-N conditions, revealing that there was a low accumulation of some intermediate products of glycolytic pathway or sucrose biosynthesis. A number of other compounds, like erythritol among others, which act as a source of precursors for carotenoids, are reduced in quantity, i.e., their biosynthesis may be modified [58]. The reduced levels of glucose, fructose, lactose, mannose and mannitol are indicative of altered metabolic and signaling function [59]. Similarly, changes in the relative levels of phenylpropanoids such as dihydroxycoumarin and phenmetrazine suggest that biosynthesis of lignin was rehabilitated [60] under low-N conditions. Metabolites showing increased response to low-N conditions in our study include raffinose, maltose, trehalose, galactinol and mannitol; this is in line with the previously described results from N-deficient *Arabidopsis* [17]. The possible reason for their increase can be explained on the basis of precursor molecules fructose and glucose, as these were detected in low concentration in plants growing under low-N conditions. The picture provided here by metabolomic profiling will serve as an important source in explaining the regulating switches of N metabolism.

Levels of flavones such as brosimacutin C and deoxymiroestrol increased under low-N conditions. One of the possible reasons for increased flavonoid synthesis under nitrogen limitation is that enhanced PAL activity will free nitrogen for amino acid metabolism, whereas carbon products are shunted via 4-coumaroyl-CoA into the flavonoid biosynthetic pathway [61]. Some peptides detected as containing N, for example, Asp Thr Gly Cys, Glu Ser Gly Cys, Ala Ala Cys Cys, Glu Trp Thr Gln, Asp Pro Gln and Trp, are highly reduced under low-N conditions in order to conserve nitrogen.

The limitation of this study is that the metabolites were identified putatively. Validation of these metabolites is required in the future.

## 4. Materials and Methods

### 4.1. Plant Cultivation, N-Deficiency and N-Restoration Treatments

Two genotypes of maize (*Zea mays* L.), PHEM-2 and HM-4, were identified as being low-N tolerant (LNT) and low-N sensitive (LNS) in our earlier study [62] through the screening of thirty-three maize genotypes that included hybrids, composites and inbreds, procured from the Directorate of Maize Research and Division of Genetics, Indian Agricultural Research Institute, New Delhi. These genotypes were raised hydroponically in glasshouse at National Phytotron Facility at the Indian Agricultural Research Institute, New Delhi, India, with the optimum temperature (30 °C/24 °C D/N), relative humidity (70%) and light (natural) conditions. The nutrient solution comprised of phosphoric acid (0.5 mM), CaCl_2_ (2.25 mM), MgSO_4_ (0.75 mM), KCl (2.4 mM), NaCl (1 mM) H_3_BO_3_ (0.05 μM), MnCl_2_ (0.01 μM), ZnSO_4_ (0. 002 μM), CuSO_4_ (0.0015 μM) NH_4_Mo_7_O_24_ (0.000075 μM) and Fe-EDTA (0.074 μM). The pH of the nutrient solution was maintained at 5.6 and the solution was continuously aerated using aquarium pumps throughout the experiment. Nitrogen was supplied in the form of NH_4_NO_3_ as per the requirement of treatments. For low-N and sufficient-N treatments, the level of N was 0.05 mM and 4.5 mM, respectively. Plants growing under low-N condition were supplied with sufficient-N (4.5 mM) for restoration of N stress on the 15th day. Leaf and root samples were collected on the third (3DR), sixth (6DR) and tenth (10DR) day of the N replete condition. Likewise, leaf and root tissues were also harvested from N sufficient plants on the parallel days to relate the metabolome profiles. Harvested leaf and root samples were quickly immersed in liquid nitrogen for freezing and stored at −80 °C for further use. Two biological replicates of each sample were taken from three different experimental sets [62].

### 4.2. Sample Preparation

The stored tissues were powdered using an ice-cold mortar and pestle with the help of liquid nitrogen for extraction of metabolites [63,64].

### 4.3. Metabolite Extraction

The procedure of extracting metabolites from roots and leaves for GC–MS analysis was followed as developed previously [65]. The powdered tissue (100 mg) was extracted with HPLC-grade solvent comprising of chilled isopropanol: acetonitrile: water (3:3:2). The samples were vortexed vigorously for 10 s, followed by incubation at 70 °C for 15 min. The samples were centrifuged at 12,000× *g* for 10 min. The supernatant was collected into a screw-top glass tube and added with 1.4 mL water and 0.75 mL chloroform. The mixture was again vortexed, followed by centrifugation for 10 min at 4000× *g*. The isopropanol/acetonitrile/water phase was dried overnight in a SpeedVac concentrator (Heto Dry Winner DW1, 0-110-20952N, Denmark) [66]. The lower phase (isopropanol/acetonitrile phase) containing lipophilic compounds was discarded. Carbonyl moieties were protected by methoximation, using 50 μL of 20 mg/mL solution of the corresponding methoxyamine hydrochloride in pyridine at 30 °C for 90 min. Subsequently, acidic protons were derivatized with 70 μL N-methyl-N-trimethylsilyl trifluoroacetamide (MSTFA) at 37 °C, respectively for 90 min [67]. The narrow range of detection of only volatile and thermally stable metabolites by GC–MS was overcome by LC–MS analysis. For LC–MS analysis, 500 mg of frozen ground tissue was placed in a 2.5 mL polyethylene screw-cap tube and frozen in a liquid nitrogen holding station (SPEX Sampleprep, Metuchen, NJ, USA) until all samples were ground. The fine powdered samples were extracted with 2.0 mL of ice-cold extraction solution (100 mL methanol acidified with 0.1% formic acid LC–MS grade *v/v*; Sigma-Aldrich, Oakville, ON, Canada). Samples were vortexed for 60 sec and kept in the icebox until all samples were prepared. The Eppendorf tubes were then sonicated in water bath at 20 °C and 40 kHz (Branson sonicator, Thomas Scientific, Swedesboro, NJ, USA) for 15 min. The mixture was then centrifuged at 20,000× *g* for 10 min and filtered through 0.2 µM PFFE, using a disposable syringe into an LC–MS autosampler vial. Optimization of sample dilutions was done in order to ensure peak intensities in the linear range and prevent detector saturation. The samples of maize genotypes were diluted 3-fold with the extraction buffer. Prior to the LC–MS analysis, the samples were allowed to equilibrate at 25 °C in the dark for 45 min and maintained in similar conditions during their analysis. The characteristic mass error was less than 4 ppm.

### 4.4. Normalization and Metabolite Identification

Putative identification of known metabolites was carried out with the aid of metabolomic libraries present in the database attached with GC–MS and LC–MS instruments, respectively (Glom, HMDB, NIST, Lipid maps and LIMS). Determination of analytical characteristics of metabolites detected by GC and LC was made possible through a number of commercially available purified standard compounds in the laboratory information management system (LIMS). The match of the specific compound or isobaric entity with the aid of these commercially available libraries depends on both the chromatographic properties and mass spectra of the sample. The variations that may occur from instrument inter-day tuning differences were corrected by normalizing the data [68]. Essentially, each compound was corrected in run-day blocks by registering the medians equal to one (1.00) and normalizing each data point proportionately.

### 4.5. PCA and Statistical Analysis for Metabolite Profiling

Data obtained from both GC–MS and LC–MS were rigorously analyzed by using MetaboAnalyst 2.02 (www.metaboanalyst.ca) web [69]. The data, in triplicates in the form of area of each putatively identified metabolite, were uploaded into the software in a comma separated excel file (CSV format). The software did an integrity check of the submitted data file automatically. Filtering data solved the prevention of errors that may occur due to mathematical transformations. Normalization of data was also done by pooling controlled groups. Large data sets were reduced by performing a principal component analysis (PCA) with the help of MeltDB [70]. Furthermore, data were log10-transformed and centered for generation of heatmaps and statistics. For metabolites detected in leaf, root and restoration samples; a one-way-ANOVA and Tukey’s test (*p* ≤ 0.05) were carried out by using SigmaPlot 11 (Systat Software USA, San Jose, CA 95131, USA). The important features identified by ANOVA analysis are available in Figure S4 and Table S4. A Multi Experiment Viewer (MeV 4.9, http://www.tm4.org/mev.html) tool was used to generate heatmaps by using the Pearson’s correlation and complete linkage. Response ratio −N/+N of altered metabolites was calculated from untransformed mean values for linking them with different metabolic pathways in order to create the metabolic map.

## 5. Conclusions

The present study revealed that nitrogen deficiency has a broad range influence on the metabolism of the leaf and root, causing a reduction in a number of organic and amino acids and increases in the level of various carbohydrates, phosphoesters and several secondary metabolites. The N-deficiency-induced alteration in the level of metabolites was confirmed by the restoration of N supply to the N-deficient condition. The limitation of this study is that the metabolites were identified putatively. Validation of these metabolites in the future study will help in identifying the metabolites with greater potential to restore in response to N restoration, which are likely to the cynosure of future studies related to N metabolism. However, the most remarkable message of the present study is that the foremost biological processes and stress-responsive regulatory factors related to carbon utilization share common characteristics, as can be visible by taking the snapshot of the whole metabolome through untargeted metabolite profiling.

## Figures and Tables

**Table 1:** Growth and physiological parameters of low-N sensitive (HM-4) and low-N tolerant (PHEM-2) genotypes of maize at sufficient (4.5 mM N) and low (0.05 mM N) nitrogen supply.

Growth and Physiological Parameters	HM-4 Genotype		PEHM-2 Genotype	
	Sufficient-N (4.5 mM)	Low-N (0.05 mM)	Sufficient-N (4.5 mM)	Low-N (0.05 mM)
Shoot length (cm/plant)	16.33a	10.07b	15.73a	15.11a
Root length (cm/plant)	23.52b	27.27a	27.47a	25.73a
Plant Biomass (g/plant)	1.23a	0.88b	1.67a	1.55b
Leaf area (cm^2^/plant)	64.7a	47.9b	96.1a	77.1b
Photosynthetic rate (µmol CO_2_ m^−2^ s^−1^)	27.3a	20.7b	29.7a	27.19b
Total Chlorophyll (mg g^−1^ FW)	1.79a	1.12b	1.86a	1.88a
NR activity (µmol g^−1^ FWh^−1^)	5.35a	3.74b	5.19a	4.75b
Concentration of nitrogen (%)	3.17a	2.07b	3.18a	2.87b
Plant N uptake (mg/plant)	38.9a	18.2b	53.10a	44.4b

Values are the mean of three independent replicates (*n* = 3). Values of the same variable within the cultivar followed by different letters are significantly different according to an ANOVA-protected Least Significant Difference 0.05 test.

**Table 2:** Results of the pathway analysis of leaf and root samples of the low-N sensitive (HM-4) and low-N tolerant (PEHM-2) maize genotypes grown under the conditions of low-N, sufficient-N and restorations of N supply (3rd, 6th and 10th day).

Metabolic Pathways	Total	Expected	Hits	Raw *p*	-LOG (*p*)	Holm Adjust	FDR *p*	Impact
Aminoacyl-tRNA biosynthesis	46	1.90	14	7.51 × 10^−10^	21.01	7.13 × 10^−8^	7.13 × 10^−8^	0.11111
Galactose metabolism	27	1.12	8	6.29 × 10^−6^	11.976	0.00059172	0.00029901	0.33624
Alanine, aspartate and glutamate metabolism	22	0.91	7	1.51 × 10^−5^	11.104	0.0014001	0.00047675	0.45324
Arginine biosynthesis	18	0.74	6	4.86 × 10^−5^	9.932	0.0044707	0.0011541	0.25729
Glyoxylate and dicarboxylate metabolism	29	1.20	6	0.00087103	7.0458	0.079263	0.01655	0.22338
Glycine, serine and threonine metabolism	33	1.36	5	0.0098897	4.6163	0.89007	0.14956	0.51346
Starch and sucrose metabolism	22	0.99	4	0.01102	4.508	0.98082	0.14956	0.50234
Cyanoamino acid metabolism	26	1.07	4	0.01991	3.9166	1	0.23643	0
Pentose and glucuronate inter conversions	17	0.70	3	0.030183	3.5005	1	0.28673	0.09524
Butanoate metabolism	17	0.70	3	0.030183	3.5005	1	0.28673	0.13636
Citrate cycle (TCA cycle)	20	0.82	3	0.046359	3.0713	1	0.39716	0.11468
Lysine biosynthesis	9	0.37	2	0.050168	2.9924	1	0.39716	0
Valine, leucine and isoleucine biosynthesis	22	0.91	3	0.05909	2.8287	1	0.43181	0
Glutathione metabolism	27	1.12	3	0.097154	2.3315	1	0.61964	0.07071
Nicotinate and nicotinamide metabolism	13	0.54	2	0.097838	2.3244	1	0.61964	0.17576
Arginine and proline metabolism	28	1.16	3	0.10573	2.2468	1	0.6278	0.22747
Sulfur metabolism	15	0.62	2	0.12502	2.0793	1	0.69862	0.03315
Tyrosine metabolism	18	0.74	2	0.16858	1.7803	1	0.88973	0.23784
Fructose and mannose metabolism	20	0.82	2	0.1989	1.615	1	0.99242	0.03695
Carbon fixation in photosynthetic organisms	21	0.87	2	0.2143	1.5404	1	0.99242	0
Isoquinoline alkaloid biosynthesis	6	0.25	1	0.22413	1.4955	1	0.99242	0.41176
Phenylalanine, tyrosine and tryptophan biosynthesis	22	0.91	2	0.22982	1.4704	1	0.99242	0.02002
Tryptophan metabolism	23	0.95	2	0.24542	1.4048	1	1	0.5862
Monobactam biosynthesis	8	0.33	1	0.28724	1.2474	1	1	0
Tropane, piperidine and pyridine alkaloid biosynthesis	8	0.33	1	0.28724	1.2474	1	1	0
Cysteine and methionine metabolism	46	1.90	3	0.29544	1.2193	1	1	0.12832
Amino sugar and nucleotide sugar metabolism	50	2.07	3	0.3419	1.0732	1	1	0
Nitrogen metabolism	12	0.49	1	0.3987	0.91954	1	1	0
Selenocompound metabolism	13	0.54	1	0.42377	0.85855	1	1	0
Valine, leucine and isoleucine degradation	37	1.53	2	0.45794	0.78102	1	1	0
Pyrimidine metabolism	38	1.57	2	0.47199	0.7508	1	1	0.02773
Purine metabolism	63	2.60	3	0.48919	0.71499	1	1	0.00383
Sphingolipid metabolism	17	0.70	1	0.51419	0.66517	1	1	0
Lysine degradation	18	0.74	1	0.53452	0.62639	1	1	0
Beta-Alanine metabolism	18	0.74	1	0.53452	0.62639	1	1	0
Ascorbate and aldarate metabolism	18	0.74	1	0.53452	0.62639	1	1	0.02239
Pentose phosphate pathway	19	0.78	1	0.55401	0.59057	1	1	0
Propanoate metabolism	20	0.83	1	0.5727	0.55739	1	1	0
Pyruvate metabolism	22	0.91	1	0.6078	0.49791	1	1	0
Thiamine metabolism	22	0.91	1	0.6078	0.49791	1	1	0
Biosynthesis of unsaturated fatty acids	22	0.91	1	0.6078	0.49791	1	1	0
Pantothenate and CoA biosynthesis	23	0.95	1	0.62427	0.47117	1	1	0
Alpha-Linolenic acid metabolism	27	1.12	1	0.68363	0.38034	1	1	0.11368
Inositol phosphate metabolism	28	1.16	1	0.69696	0.36102	1	1	0
Ubiquinone and other terpenoid-quinone biosynthesis	35	1.45	1	0.77603	0.25356	1	1	0
Phenylpropanoid biosynthesis	35	1.45	1	0.77603	0.25356	1	1	0

Total = total number of compounds in the pathway; Hits = actual matched number from the user uploaded data; Raw *p* = original *p* value calculated from the enrichment analysis; Holm *p* = the *p* value adjusted by the Holm–Bonferroni method; FDR *p* = the *p* value adjusted using the false discovery rate; Impact = pathway impact value calculated from the pathway topology analysis.

## Supplementary Materials

The following are available online at https://www.mdpi.com/2223-7747/9/11/1459/s1, Figure S1: 15-day-old low-N sensitive (HM-4) and low-N tolerant (PEHM-2) maize cultivars growing under low N (0.05 mM) and sufficient N (4.5 mM) conditions, Figure S2: Impact of low N on secondary metabolites and their putative identification as metabolic markers using random forest, Figure S3: Metabolic responses to N deficiency in leaves and roots of low-N tolerant (PEHM-2) and low-N sensitive (HM-4). The calculated data shown above is log transformed fold change (0.05mM /4.5mM N) of selected leaf and root metabolites detected by GC-MS in low N (0.05mM), sufficient N (4.5mM) and recovery (3DR, 6DR, 10DR) samples. Level of significance between sufficient N (4.5mM) and low N (0.05mM) treatments was tested by the Student’s *t*-test (*p*-value < 0.05). Significantly changed metabolites in various groups are represented in bolded form. Up regulation and down regulation of metabolites is indicated with the help of color bar scale, up regulated (green) or down regulated (red) in metabolite logarithmic fold change as indicated in the color index (*n* = 8), Figure S4: The red marked circles represent significantly changing metabolites identified by ANOVA, Table S1: Principle Component Analysis of data of metabolites of root and leaves of maize genotypes under the conditions of low N, sufficient N and restoration of N, Table S2: Random Forest data for putative identification of metaboloic markers, Table S3: List of metabolites detected as known metabolites with aid of METLIN tandem database (Scripps Center for Metabolomics), Table S4: List of metabolites which showed significant change under low N (0.05mM) conditions (Identified by ANOVA).

## Abbreviations

NUE	Nitrogen Use Efficiency
GC–MS	Gas Chromatography-Mass Spectrometry
LNT	Low-N tolerant (PEHM-2)
LNS	Low-N sensitive (HM-4)
HMW	High Molecular Weight Compounds
LMW	Low Molecular Weight Compounds
PCA	Principle component Analysis
PC1	Principle Component 1
FDR	False Discovery Rate
